# Adverse childhood experiences and suicidal ideation among immigrants in Santiago, Chile

**DOI:** 10.1192/j.eurpsy.2021.1087

**Published:** 2021-08-13

**Authors:** A. Errazuriz, D. Avello, S. Morales, R. Pino

**Affiliations:** Psychiatry, School Of Medicine, Pontificia Universidad Catolica de Chile, Santiago, Chile

**Keywords:** immigrant, healthy immigrant effect, adverse childhood experiences, Suicidal ideation

## Abstract

**Introduction:**

Understanding suicidal ideation and its association with childhood adversity is crucial for preventing suicide. Although the “healthy immigrant effect”, whereby immigrants are healthier than the native-born population, has been well documented across studies, little research has examined the presence of such effect on lifetime suicidal ideation (LSI) and its association to early adversity.

**Objectives:**

The aim of this study was to compare the prevalence of LSI between the immigrant and native-born population in Chile and explore the association between childhood adversity and suicidal ideation in immigrants.

**Methods:**

Data from two cross-sectional health surveys: the Santiago Immigrant Wellbeing Study (STRING, n=1,091; 2019) and the Chilean National Health Survey (ENS2016, n=3,432) were used. Each study used multistage probability sampling and estimates were weighted to approximate the distribution of demographic variables in each population. Outcomes included LSI measured by WHO-CIDI and an adapted version of the Adverse Childhood Experience Questionnaire. Multivariate logistic regression was employed.

**Results:**

indicated that immigrants were less likely to report LSI compared with the native-born population. Moreover, male and female immigrants had lower risk of having SI than native-born counterparts. After controlling for socioeconomic status, social support, and health conditions, childhood adversities predicted an increased risk of LSI in immigrants. No gender differences were found in the effects of childhood adversity on suicidal thoughts.

**Conclusions:**

Findings confirm the presence of a healthy immigrant effect in LSI and support a life course perspective, highlighting the importance of assessing early life disadvantages to understand suicidal ideation among immigrants.

